# Crohn’s Disease: Radiological Answers to Clinical Questions and Review of the Literature

**DOI:** 10.3390/jcm13144145

**Published:** 2024-07-16

**Authors:** Laura Maria Minordi, Francesca Bice D’Angelo, Giuseppe Privitera, Alfredo Papa, Luigi Larosa, Lucrezia Laterza, Franco Scaldaferri, Brunella Barbaro, Luigi Carbone, Daniela Pugliese

**Affiliations:** 1 UOC di Radiologia Addomino-Pelvica, Dipartimento di Diagnostica per Immagini, Radioterapia Oncologica ed Ematologia, Fondazione Policlinico Universitario “A. Gemelli” Istituto di Ricovero e Cura a Carattere Scientifico (IRCCS), Largo Francesco Vito, 1, 00168 Roma, Italy; francescabice.dangelo01@icatt.it (F.B.D.);; 2 Dipartimento di Scienze della Salute, Università degli Studi di Milano, 20122 Milan, Italy; gpp.privitera@icloud.com; 3 Unità Operativa Semplice Dipartimentale Day Hospital (UOSD DH) Medicina Interna e Malattie dell’Apparato Digerente, Fondazione Policlinico Universitario “A. Gemelli” Istituto di Ricovero e Cura a Carattere Scientifico (IRCCS), Università Cattolica del Sacro Cuore, 00168 Roma, Italy; alfredo.papa@unicatt.it; 4 CEMAD Digestive Disease Center, Fondazione Policlinico Universitario “A. Gemelli” Istituto di Ricovero e Cura a Carattere Scientifico (IRCCS), Catholic University of Rome, 00168 Rome, Italy; lucrezia.laterza@policlinicogemelli.it (L.L.); franco.scaldaferri@policlinicogemelli.it (F.S.); daniela.pugliese@policlinicogemelli.it (D.P.); 5 Dipartimento di Medicina e Chirurgia Traslazionale, Università Cattolica del Sacro Cuore, L. Go A. Gemelli 8, 00168 Rome, Italy; 6 UOC Pronto Soccorso, Medicina d’Urgenza e Medicina Interna, Ospedale Isola Tiberina Gemelli Isola, 00186 Rome, Italy; luigi.carbone@policlinicogemelli.it; 7 UOS Gastroenterologia, Ospedale Isola Tiberina Gemelli Isola, 00186 Rome, Italy

**Keywords:** inflammatory bowel disease, Crohn’s disease, Computed Tomography, Magnetic Resonance Imaging

## Abstract

**Background:** Crohn’s disease (CD) is a chronic, progressive inflammatory condition, involving primarily the bowel, characterized by a typical remitting–relapsing pattern. Despite endoscopy representing the reference standard for the diagnosis and assessment of disease activity, radiological imaging has a key role, providing information about mural and extra-visceral involvement. **Methods:** Computed Tomography and Magnetic Resonance Imaging are the most frequently used radiological techniques in clinical practice for both the diagnosis and staging of CD involving the small bowel in non-urgent settings. The contribution of imaging in the management of CD is reported on by answering the following practical questions: (1) What is the best technique for the assessment of small bowel CD? (2) Is imaging a good option to assess colonic disease? (3) Which disease pattern is present: inflammatory, fibrotic or fistulizing? (4) Is it possible to identify the presence of strictures and to discriminate inflammatory from fibrotic ones? (5) How does imaging help in defining disease extension and localization? (6) Can imaging assess disease activity? (7) Is it possible to evaluate post-operative recurrence? **Results:** Imaging is suitable for assessing disease activity, extension and characterizing disease patterns. CT and MRI can both answer the abovementioned questions, but MRI has a greater sensitivity and specificity for assessing disease activity and does not use ionizing radiation. **Conclusions:** Radiologists are essential healthcare professionals to be involved in multidisciplinary teams for the management of CD patients to obtain the necessary answers for clinically relevant questions.

## 1. Introduction

Inflammatory bowel diseases (IBDs) are chronic, progressive inflammatory diseases, involving primarily the bowel, characterized by a typical remitting-relapsing pattern. The aetiology is unknown, but the most accredited hypothesis is that environmental factors induce in genetically predisposed hosts an alteration of intestinal microbiota, gut epithelial barrier leaks and a subsequent dysregulation of the gut immune response, thus being responsible for bowel damage [1].

The incidence of IBD is quite stable in western countries, but the prevalence is progressively growing due to the low rate of disease-related mortality (stage of compounding prevalence) [2]. IBD is commonly diagnosed at a young age, with a peak of incidence between 18 and 35 years, even though it is not rare a diagnosis among paediatric and elderly populations.

Two main forms are recognized: Crohn’s disease (CD), potentially affecting all gastrointestinal tracts and characterized by a transmural inflammation, and ulcerative colitis (UC), involving only the colon at the mucosa layer. If bloody diarrhoea represents the typical symptom of UC, the spectrum of clinical manifestations for CD can be quite variable. The evolution of chronic inflammation can progressively lead to the development of complications in both conditions, such as stenosis or fistulas for CD, and luminal narrowing or colorectal cancer for UC [3,4]. Endoscopy is the reference standard for the diagnosis and assessment of disease activity in both diseases, but imaging has a key role as well, providing information about parietal and extra-visceral involvement. In particular, imaging is useful for the diagnosing and staging of small bowel CD, while its role in UC is limited to the presence of complications or in cases of acute severe presentation [5].

In this setting, a strict collaboration between radiologists and gastroenterologists/surgeons is required to improve imaging performance, and radiologists are asked to answer specific clinical questions. The aim of this paper was to describe the contribution of imaging in the management of CD by answering to seven practical clinical and/or surgical questions.

## 2. Methods

### 2.1. Questions

We established 7 practical and clinically relevant questions which we tried to provide answers to, in detail:

(1) What is the best technique for the assessment of small bowel CD? (2) Is imaging a good option to assess colonic disease? (3) Which disease pattern is present; inflammatory, fibrotic or fistulizing? (4) Is it possible to identify the presence of strictures and to discriminate inflammatory from fibrotic ones? (5) How does imaging help defining disease extension and localization? (6) Can imaging assess disease activity? (7) Is it possible to evaluate post-operative recurrence?

### 2.2. Search on Medline Electronic Database

Accordingly, we conducted a comprehensive electronic search on the Medline electronic database through January 2024 with no language restrictions using the following search terms: (“IBD”) OR (“Crohn’s Disease (CD)”) AND (“imaging”) or (“radiology”) or (“Computed Tomography”) or (“Magnetic Resonance”). Two independent reviewers (LMM and LL) independently evaluated the title and abstract of studies identified in the primary search and then the full text of selected articles. Papers were selected based on their capability to provide evidence relevant to our pre-specified questions.

## 3. What Is the Best Technique for the Assessment of Small Bowel CD?

**Figure 1:**
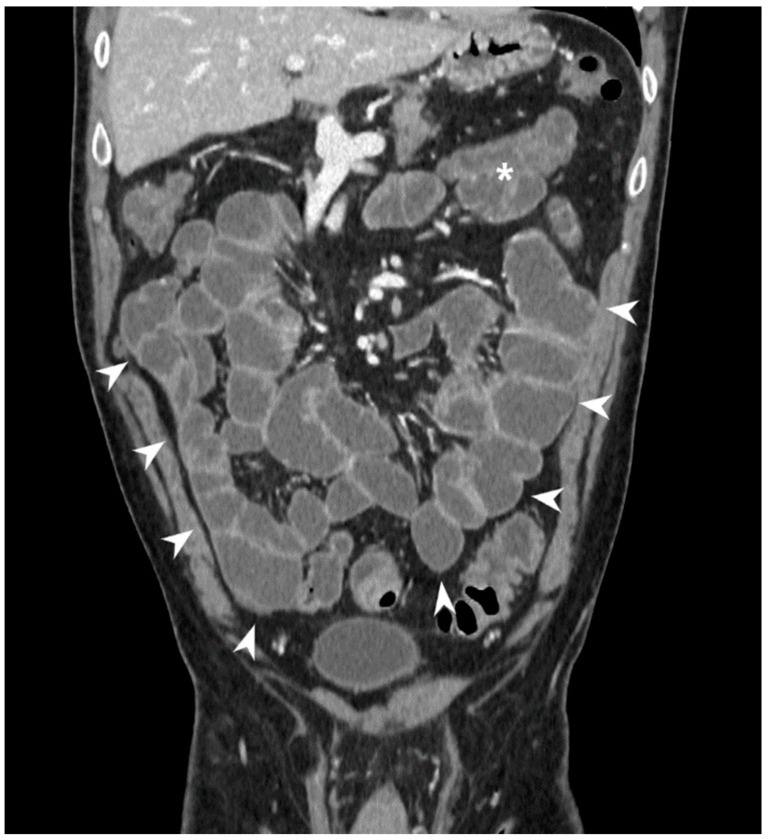
Coronal CT-E image after iodinated contrast medium injection: example of optimal distention of the ileal loops obtained by oral administration contrast medium (arrowheads); jejunal loops are on the top left (white asterisk). Original figures by LMM and LL.

Computed Tomography (CT) and Magnetic Resonance Imaging (MRI) are the most frequently used radiological techniques in clinical practice for both the diagnosis and the staging of CD involving the small bowel in non-urgent settings. However, for patients presenting to the emergency room for acute abdominal pain, potentially related to an intestinal obstruction or sepsis, an abdominal CT scan with iv contrast medium and without distension of the intestinal loops is recommended [6,7]. The CT and the MRI must be performed with a rigorous technique: in particular, it is essential to have optimal distention of the intestinal loops. Accordingly, they are both performed with the administration of a contrast medium by mouth (MR-enterography, MR-E; CT-enterography, CT-E) or by naso-jejunal tube (MR-enteroclysis, MR-e; CT-enteroclysis, CT-e). The administration of the contrast medium through a naso-jejunal tube allows to obtain a better distension of the jejunum, which is often collapsed during image acquisition when the medium is administered orally [8,9,10,11] (Figure 1). 

Despite these differences in the distention of the intestinal loops, both methods of contrast administration have shown similar diagnostic accuracy for the diagnosis of CD and its complications, but patients’ discomfort can be considerably higher using CT/MR-e. The values of diagnostic imaging’s accuracy range from 95% to 100% for MR-e and from 72% to 98% for MR-E [12,13,14,15,16]. 

Accordingly, CT/MR-E are the most frequently used radiological techniques in clinical practice [12]. Many contrast agents are used to distend the intestine in both CT-E and MR-E, such as polyethylene glycol solution (PEG), oil emulsions, water, air, Mucofalk^®^, dilute barium sulphate, mannitol, sorbitol, and locust bean gum (Table 1); PEG is one of most commonly administered, due to its low cost and few side effects.

**Figure 2:**
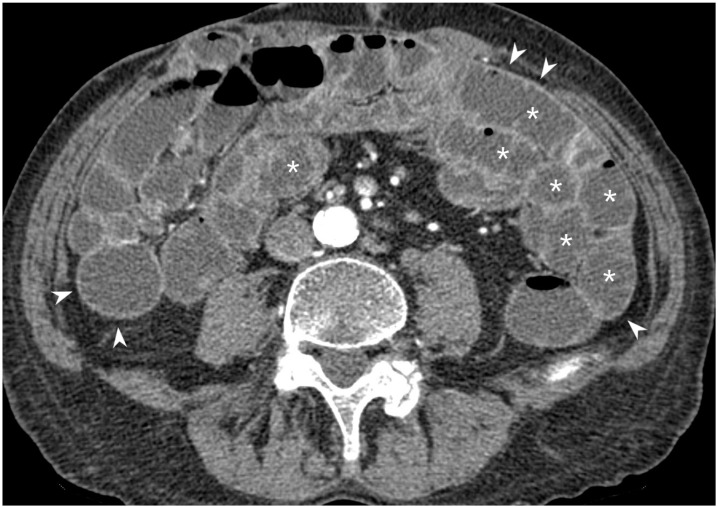
Axial CT-E image after iodinated contrast medium injection: example of intestinal loops’ distension by a hypodense contrast medium (polyethylene glycol solution), showing the hypodensity of the jejunal lumen (asterisks) and the hyperdensity of the intestinal wall (arrowheads). Original figures by LMM and LL.

The loops can be considered adequately distended when good distension of the lumen and clear visualization of the small bowel wall are obtained; the distension can be classified into five degrees: 1, collapse; 2, distension less than 50% of the adequately distended segment; 3, distension > 50% but <80%; 4, distension in the 80–100% range; and 5, optimal distension. Use of antimotility drugs helps to reduce bowel movements that can mimic a wall thickening. These contrast agents make the lumen hypodense in CT and allow a better visualization of the small bowel internal wall and the degree of enhancement after intravenous injection of the iodinated contrast medium (Figure 2). 

**Figure 3:**
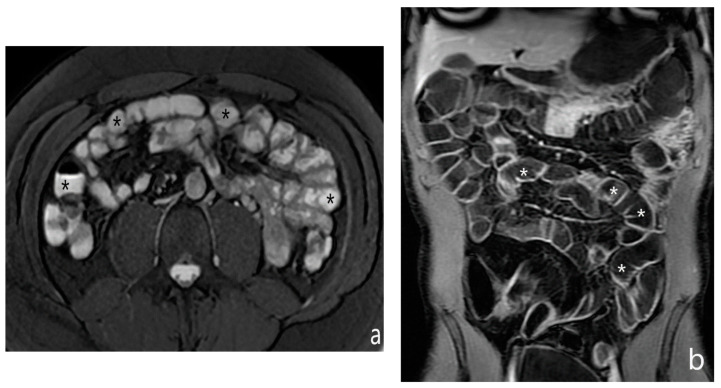
MR-E: example of intestinal loops’ distention by biphasic contrast medium (polyethylene glycol solution). Intestinal lumen (asterisks) is hyperintense in T2-weighted axial image (**a**) and hypointense in T1-weighted coronal image after gadolinium injection (**b**). Original figures by LMM and LL.

Conversely, in MRI, the appearance depends on the specific sequences of acquisition: on T2-weighted images, the intestinal lumen is hyperintense, while T1-weighted sequences after injection of gadolinium contrast medium allow to visualize the degree of enhancement of the bowel wall, thanks to the contrast between the dark bowel lumen and the hyperintense intestinal walls (Figure 3).

**Figure 4:**
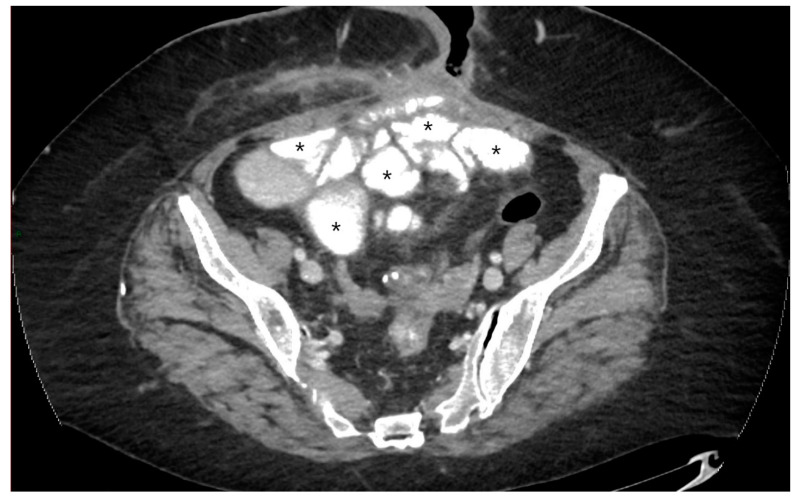
CT-E axial image: example of intestinal distension by oral administration of hydrosoluble iodine contrast medium in patient who has undergone intestinal resection with suspected post-surgical fistula. The image shows the hyperdense appearance of the ileal lumen (asterisks). Original figures by LMM and LL.

In CT, even if the hyperdense contrast medium (1–2% barium sulphate suspension or a 2–3% water-soluble iodinated solution) hides the normal intestinal wall and the characteristics of its enhancement, it can be used in patients with suspected intestinal perforation or fistulizing disease, as it allows better visualization of contrast medium extravasation and the fistulas (Figure 4) [17,18,19].

CT-E is still the most widely used technique; however, whenever possible, MR-E should be preferred due to the absence of ionizing radiation and the greater contrast resolution, which allows for a better visualization of the intestinal wall and its fibrotic and/or inflammatory alterations [20,21]. In fact, during their lifetime, almost 10% of patients affected by CD are exposed to a potentially harmful quantity of radiation, established as ≥50 milli-sieverts (mSv) (corresponding to five CT abdomen examinations) [22]. However, radiation exposure may significantly differ from one model of CT to another; for example, a multidetector-CT allows a reduction of radiation exposure up to 10–60%, thanks to effective detector conformation, image postprocessing algorithms, better filters, and automatic exposure controls [23]. Moreover, several techniques have been developed to reduce the radiation dose, such as moderation of exposure time, voltage, amperage, the use of noise-reduction filters and/or of concentrated oral contrast (Telebrix 9% instead of the commonly used 3% concentration) and of high noise index (MBCT—modified small bowel CT), and the absence of the administration of intravenous (iv) contrast [24]. These low-dose techniques can provide precise and useful diagnostic information, despite resulting in lower quality images [25].

## 4. Is Imaging a Good Option to Assess Colonic Disease?

**Figure 5:**
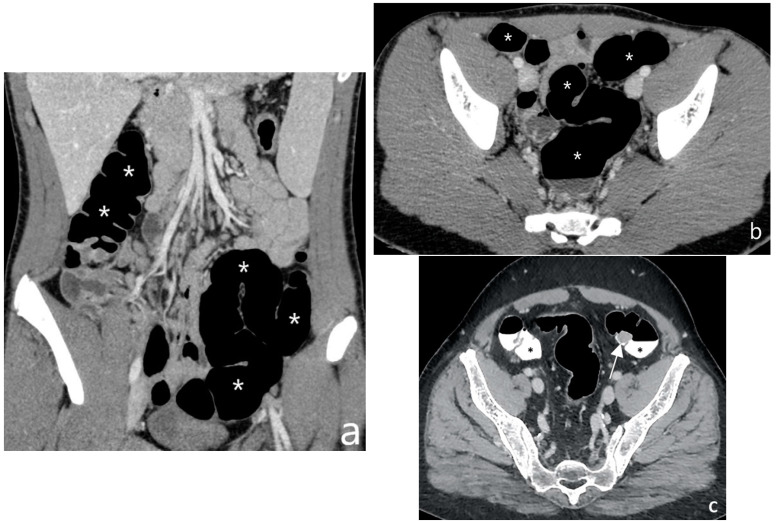
CT after iodinated contrast injection: example of colon distension using air by endorectal insufflation in the coronal (**a**) and axial (**b**) planes (asterisks). (**c**) Another example of colonic distension using air by endorectal insufflation in the axial plane shows a polypoid thickening of the sigmoid colon (arrow); hyperdense contrast agent for faecal tagging is also evident in the lumen (black asterisks). Original figures by LMM, LL and BB.

**Figure 6:**
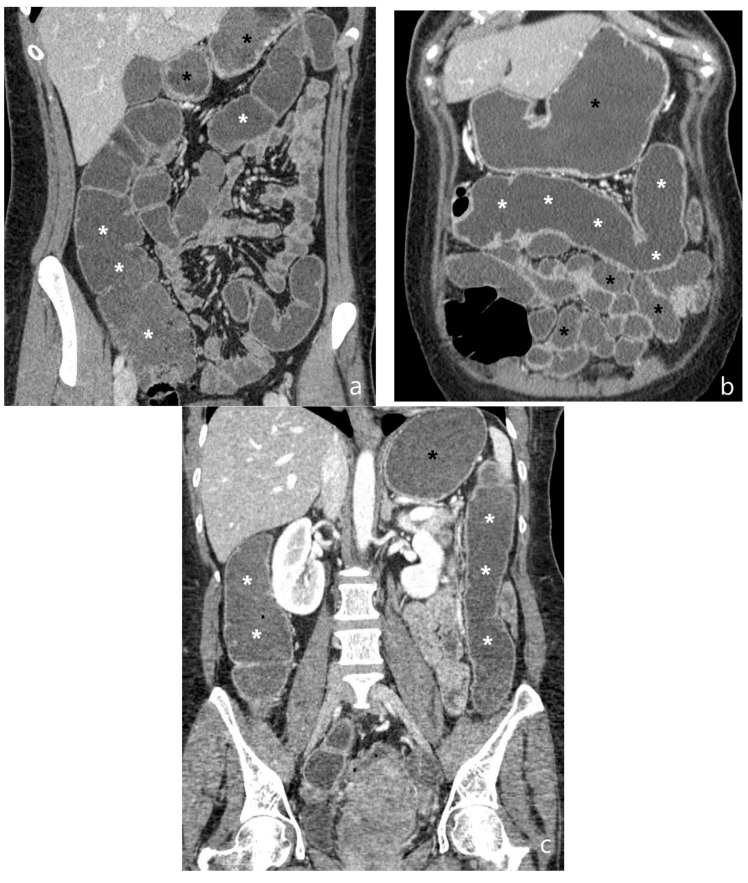
Hydro-CT. Coronal (**a**–**c**) images show good distension of the colon obtained by both endorectal (white asterisks) and oral (black asterisks) administration of water and hypodense contrast medium (polyethylene glycol solution). Original figures by LMM and LL.

CT-E and MR-E are usually performed for the evaluation of the small intestine, while endoscopy is preferred when it comes to colonic disease. Even though colonic distention is not routinely recommended, in specific clinical situations—such as the presence of colonic strictures—it could be helpful [26]. Bowel distension can be performed at the end of CT/MR-enterography, using air (colonography-CT) (Figure 5) or water (CT/MR-enterography with water enema or Hydro-CT/MRI) (Figure 6). 

Some studies have shown that the distention of the colon through the rectum improves the visualization of the ileo-caecal region in CT-E, thereby increasing the diagnostic accuracy of the exam, although the patient’s discomfort can be considerably higher. Diagnostic accuracy values with and without colonic distension were, respectively, 92% and 81% [27]. Similar findings also emerged for MR-E, since an additional rectal enema seems to increase the confidence of radiologists in the diagnosis of bowel disease, either located in the colon or in the ileocecal region [28].

Finally, a study performed in CD patients and healthy volunteers showed that performing a synchronous colonography and MR-E guaranteed a good distension of the jejunum in around 80% and of the terminal ileum in >94% of patients in both groups [29].

## 5. Which Disease Pattern Is Present: Inflammatory, Fibrotic or Fistulizing?

From a radiological point of view, CD patterns can be classified into three categories: active with predominant inflammatory signs, fibro-stenotic and fistulizing/penetrating [30]. This distinction is crucial because it can influence the therapeutic approach, specifically in the choice between medical and surgical treatment.

### 5.1. Active Subtype

In the active subtype, the typical MR/CT-E findings are the presence of bowel thickening, oedema, mucosal ulcers, segmental parietal hyperenhancement and/or stratified contrast enhancement (CE) after the administration of iv contrast [31,32].

Thickening of the intestinal wall, measured after the distension of the loop by oral contrast medium, is considered one of the most important signs of active disease and it is found in 82% of patients with CD. The normal intestinal wall appears on CT/MR-E as a hyperdense/hyperintense line with a thickness of less than 3 mm. In the case of wall thickening, it needs to be measured at the point of greatest thickness [33]. The thickening is considered minimal if between 3 and 5 mm, moderate between 6 and 9 mm, and marked if equal to or greater than 10 mm. Some studies have shown that the degree of wall thickening correlates with disease activity: thresholds of 6 mm and 10 mm have been proposed to discriminate between mild and moderate activity, and between moderate and severe activity, respectively [28,29].

**Figure 7:**
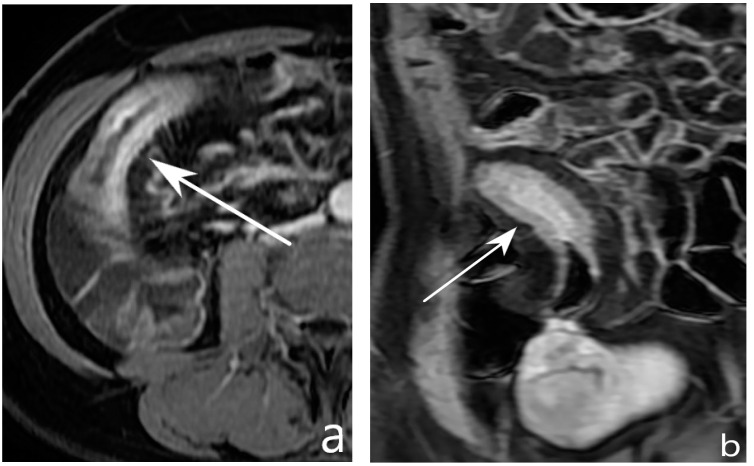
Assessment of disease activity. MR-E after gadolinium injection and administration of polyethylene glycol solution: axial (**a**) and coronal (**b**) images show pathological thickening of the distal ileum (arrow) with hyperintensity of the inner layer (mucosa) referred to hyperaemia, hypointensity of the intermediate layer (submucosa) referred to oedema, and hyperintensity of outer layer (serosa) referred to hyperaemia. Original figures by LMM and LL.

**Figure 8:**
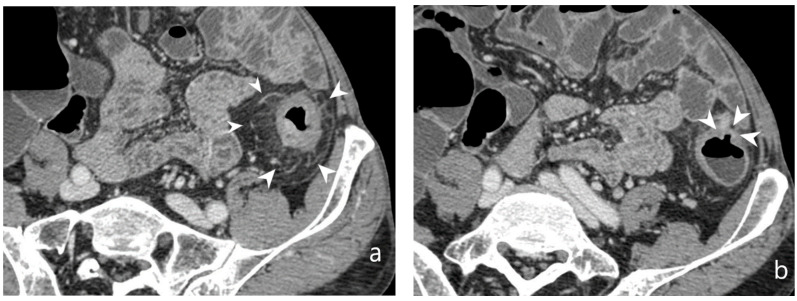
Assessment of disease activity. Axial CT-E after iodinated contrast injection and administration of polyethylene glycol solution (**a**,**b**) shows thickening of the descending colon with perivisceral fat stranding surrounding the pathological segment (white arrowheads in (**a**)). A bowel wall ulcer is also evident in another plane (white arrowheads in (**b**)). Original figures by LMM and LL.

**Figure 9:**
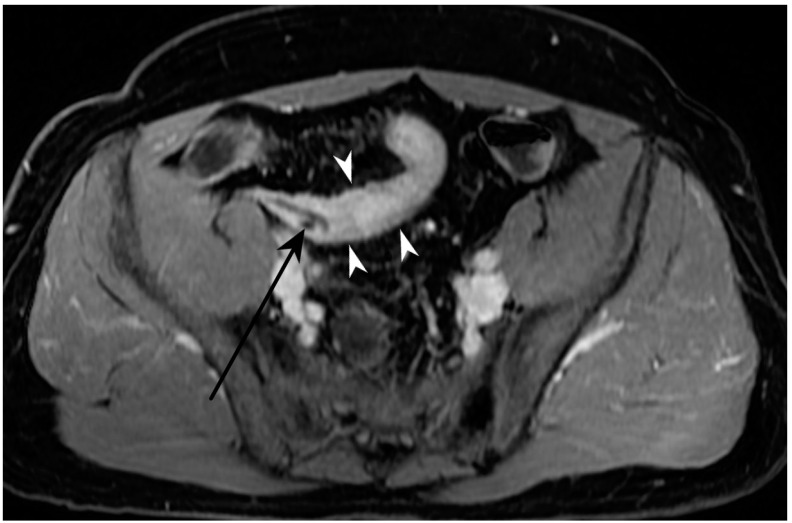
Assessment of disease activity. Axial MR-E after gadolinium injection image and administration of polyethylene glycol solution shows ileal thickening with stratified contrast enhancement (white arrows); a «minus» spot is present in the intestinal wall, indicative of deep parietal ulcer (black arrow). Original figures by LMM and LL.

In MR-E, intestinal wall oedema is detected on T2-weighted sequences and appears as a mural hyperintense signal (compared to the signal of the skeletal muscle), more evident in fat-saturation sequences [34,35]; conversely, in CT-E, wall oedema appears as a hypodense layer of the intestinal wall, after the administration of iodinated contrast medium. Stratified CE is present when mural thickening shows a marked enhancement of the inner and outer layers (i.e., the mucosa, and the muscle layer and serosa), as a consequence of hyperaemia, while the intermediate layer (i.e., the submucosa) appears hypointense/hypodense, respectively, in CT/MR-E, due to oedema. Mucosal ulcers are usually seen in the presence of more severe inflammation, and they appear as mucosal irregularities on T2 sequences in MR-E. Finally, another frequent sign of activity is the engorgement of vasa recta, related to the hyperaemia of the near mesentery, manifesting as mesenteric arterial dilation, tortuosity, prominence and wide spacing [31]. Examples of active CD are shown in Figure 7, Figure 8 and Figure 9.

### 5.2. Fibro-Stenotic Subtype

In this subtype, mural thickening is usually minimal (3–5 mm), without oedema of the bowel wall, with a homogenous, non-stratified CE after iv contrast medium administration. On MR-E, the intestinal wall shows a hypointense signal on T2-weighted sequences. In the case of stenosis, a pre-stenotic dilatation can be associated, ranging from minimal (3–4 cm) to severe (>4 cm) [33,36].

**Figure 10:**
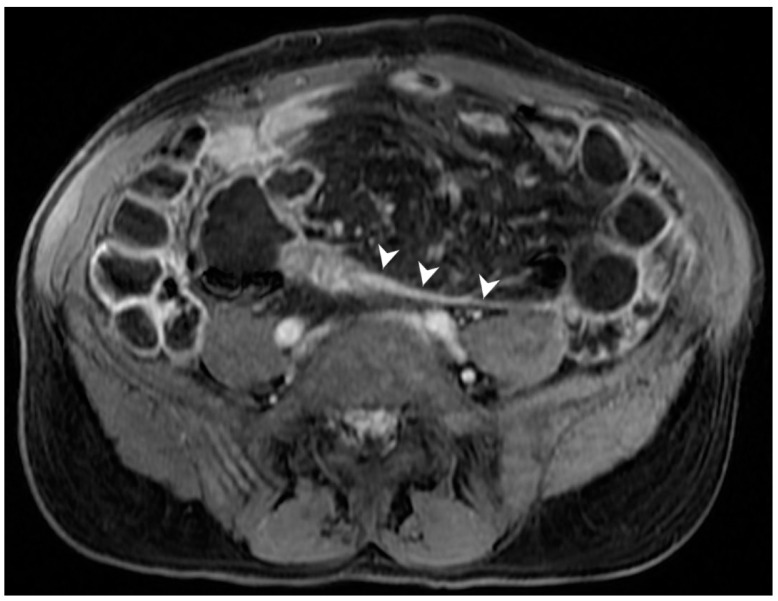
Fibro-stenotic subtype. MR-E after gadolinium injection and administration of polyethylene glycol solution, axial image shows fibrotic ileal loop with a stretched appearance (white arrowheads). Original figures by LMM and LL.

An example of fibro-stenotic disease is shown in Figure 10.

### 5.3. Fistulising/Perforating Subtype

**Figure 11:**
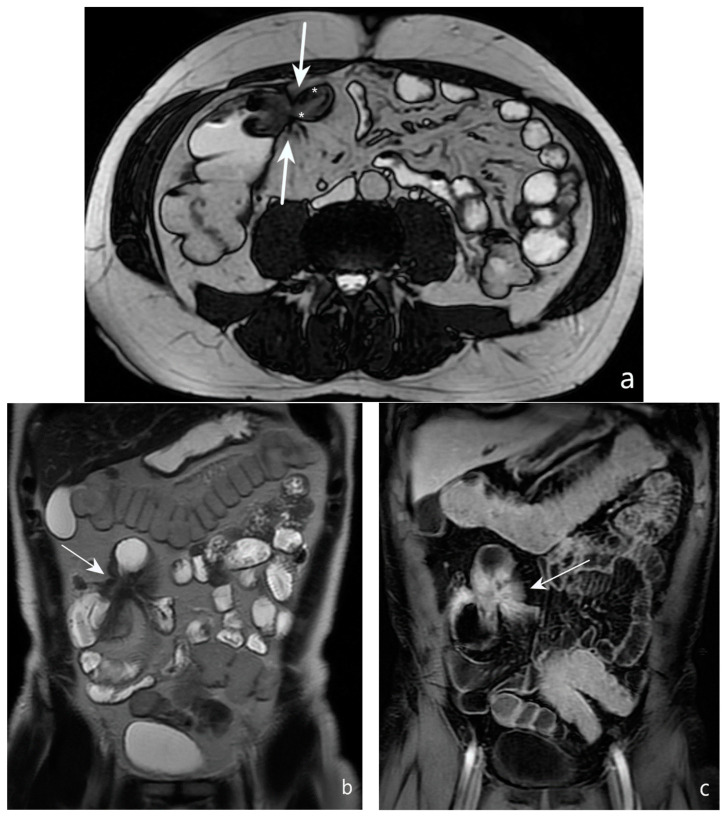
Fistulizing disease. MR-E after administration of polyethylene glycol solution; the T2-weighted axial image (**a**), T2-weighted coronal image (**b**) and contrast-enhanced fat-sat T1-weighted coronal image (**c**) show an ileo-ileal fistula in the distal ileum (arrows); focal fatty depositions in the submucosal layer are evident in a (asterisks). Original figures by LMM and LL.

**Figure 12:**
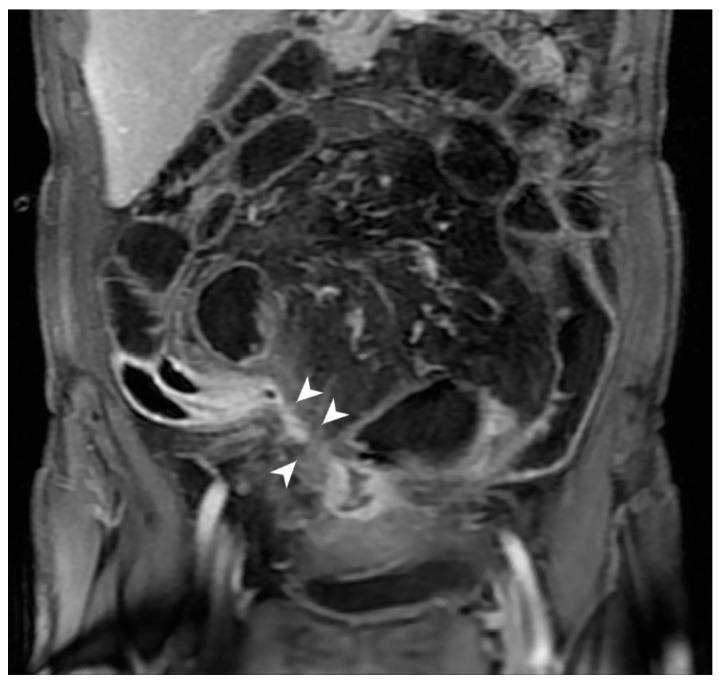
Fistulizing disease. MR-E after gadolinium injection and administration of polyethylene glycol solution, coronal image shows pathological intestinal loops in hypogastrium-right iliac fossa, with entero-enteric fistulas (white arrowheads). Original figures by LMM and LL.

**Figure 13:**
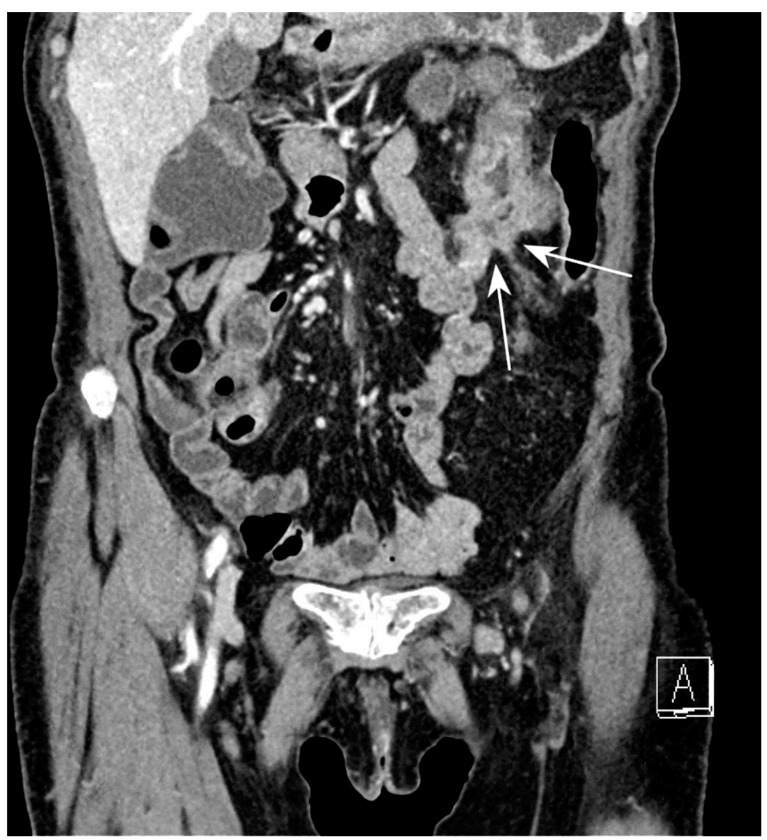
Entero-enteric fistula: CT-E coronal image after iodinated contrast injection and administration of polyethylene glycol solution shows entero-enteric fistula between the descending colon and an adjacent ileal loop (arrows). Letter “A” indicates the coronal view of the CT-E image. Original figures by LMM and LL.

This subtype is characterized by the presence of sinus tracts, fistulas and/or inflammatory masses. Sinus tracts are blind-ending tracts that develop when inflammation extends across the serosa layer. When a communication with another structure is established, the sinus tract generates a fistula. In radiological reports, a fistula is reported by describing the bowel loop of origin and the structure to which is connected (e.g., entero-enteric, entero-colic, entero-cutaneous or entero-vesical fistulas) (Figure 11, Figure 12 and Figure 13). 

**Figure 14:**
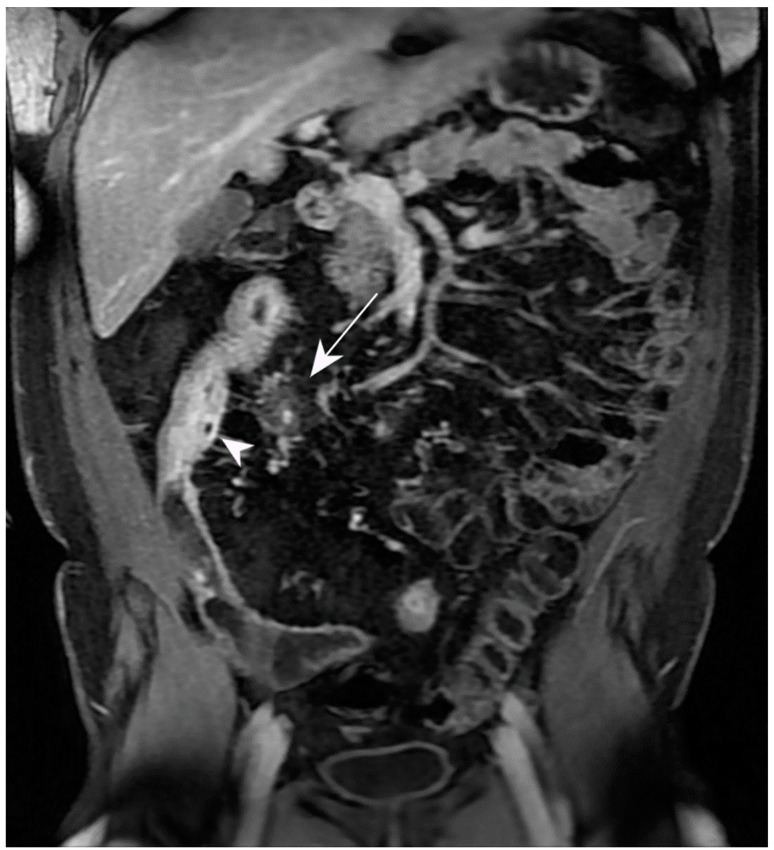
Intraparietal abscess and mesenteric inflammation. MR-E after gadolinium injection and administration of polyethylene glycol solution: axial image shows nodular formation with hypointense central core and hyperintense peripheral rim in the wall of a pathological loop (intraparietal abscess, arrowhead). In the adjacent mesentery, hyperintensity of the mesenteric fat is observed with local hypervascularization (hypertrophy of vasa recta, white arrow). Original figures by LMM and LL.

**Figure 15:**
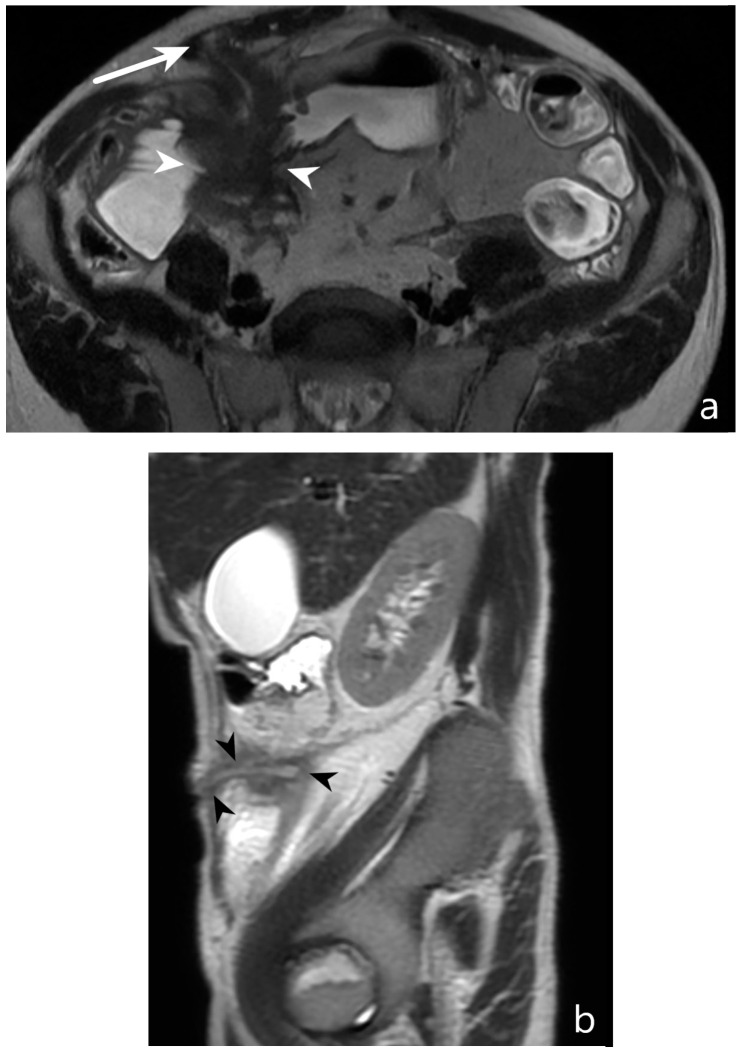
Entero-enteric fistula and entero-cutaneous fistula: MR-E after administration of polyethylene glycol solution. T2-weighted axial image (**a**) shows pathological loops with entero-enteric fistulas (white arrowheads). Further fistula with hyperintense T2 signal can be observed between the pathological loop and the anterior abdominal wall (arrow). T2-weighted sagittal image (**b**) shows the fistula between the pathological loop and the anterior abdominal wall; hyperintensity of signal in the lumen of fistula is present (black arrowheads). Original figures by LMM and LL.

**Figure 16:**
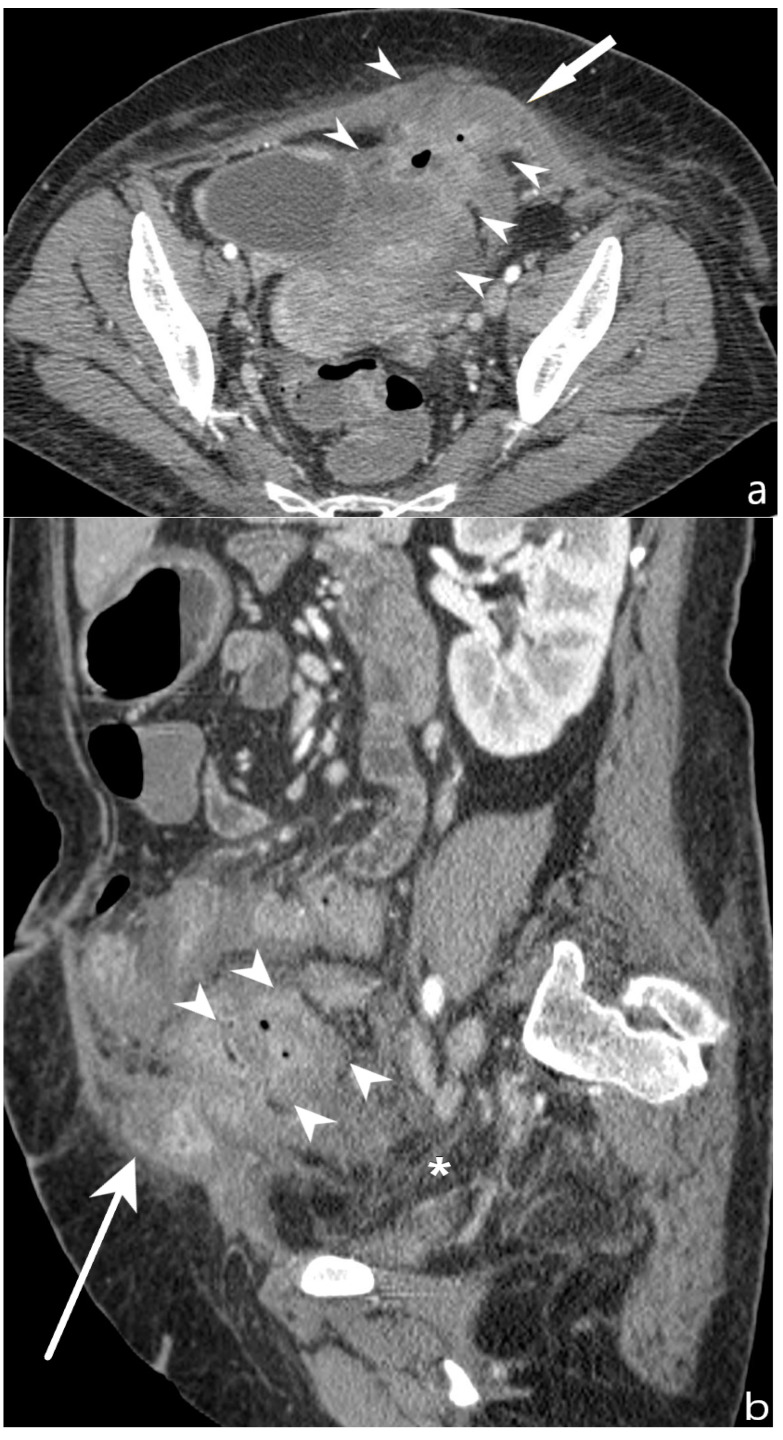
Abscess/phlegmon: CT-E after iodinated contrast injection and administration of polyethylene glycol solution. Axial image (**a**) shows an abscess/phlegmon (arrowheads) with extension to the wall of the left anterior rectus muscle (white arrow). Sagittal image (**b**) shows the extension of the abscess/phlegmon (arrowheads) to the wall of the left anterior rectus muscle (white arrow); perienteric fat stranding is present in pelvis (asterisk). Inhomogeneous hypodense tissue portion is present with anti-slope air components. Original figures by LMM and LL.

Furthermore, a consequence of fistulising disease can be the formation of an abscess, appearing as a fluid collection delimitated by an enhanced wall and, in some cases, containing air (Figure 14, Figure 15 and Figure 16). The accuracy of CT and MR for the detection of fistulas is similar [36].

## 6. Is It Possible to Identify the Presence of Strictures and to Discriminate Inflammatory from Fibrotic Ones?

### 6.1. Definition of Stricture

Currently, there is no agreement in the literature concerning the radiological definition of stricture [37,38,39,40,41,42,43].

**Figure 17:**
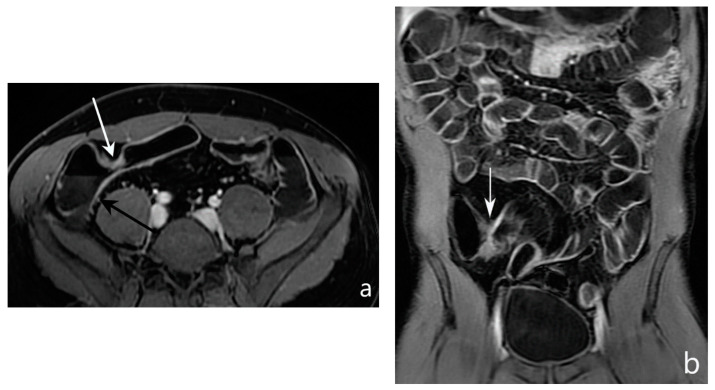
Fibro-stenotic subtype. MR-E after gadolinium injection and administration of polyethylene glycol solution; axial (**a**) and coronal (**b**) images show a homogeneous enhanced intestinal wall with narrowing of the terminal ileum (white arrow) and with dilation of the upstream small bowel loop (black arrow). Original figures by LMM and LL.

**Figure 18:**
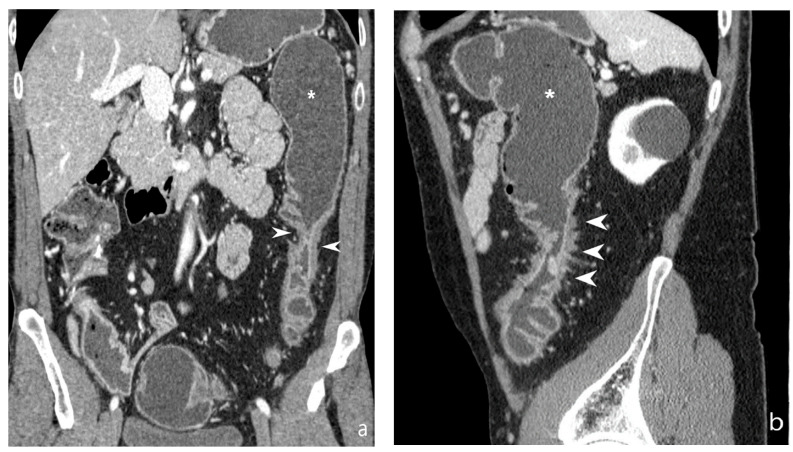
Intestinal stenosis: CT-E after iodinated contrast injection and administration of polyethylene glycol solution; coronal image (**a**) and sagittal image (**b**) show tight stenosis of the descendent colon (arrowheads), with stratified contrast enhancement; mild overdistention of the upstream descending colon (asterisk) is evident. Original figures by LMM and LL.

In 2019, Bettenworth et al. [37] performed a systematic review of radiology studies, showing great heterogeneity of the definitions adopted, which included one or more of the following features: pre-stenotic dilatation, wall thickening and/or luminal narrowing. The accuracy of cross-sectional imaging in the identification of strictures differs among studies, depending on the definition adopted—specifically, whether strictures are identified by the presence of the three abovementioned features, or just one or two. Chiorean et al. [38] used one item (bowel lumen narrowing) for stricture diagnosis in CT exams and found a sensitivity of 92% and specificity of 39%; on the contrary, another study with CT using two items (bowel lumen narrowing and increased wall thickness) found 100% sensitivity and 100% specificity [39]. Of four studies reporting the accuracy of MR-E, none provided an exact definition of stricture [40,41,44,45]. Example of stricture are shown in Figure 17 and Figure 18. 

### 6.2. Differentiation between Fibrotic or Inflammatory Stenosis

Once the presence of stenosis has been established, radiologists are called upon to define whether it is fibrotic or inflammatory. To better characterize the implications of different radiological findings, several studies have assessed the correlation between CT or MR-E findings and pathological specimens in patients who underwent intestinal resection. Adler et al. [46] found significant correlations between CT-E findings and pathological specimens in detecting both inflammatory and fibrosis signs; in particular, CT-E findings of mesenteric hypervascularity, mucosal hyperenhancement, and mesenteric fat stranding predicted the presence of inflammatory features. Chiorean et al. [38] compared CT-E signs to histopathological specimens, using a four-grade scale for inflammation (none, mild, moderate and severe) and a three-grade scale for fibrosis (none, mild/moderate and severe): in their work, CT-E correctly detected inflammation and fibrosis with a sensitivity of 77% and 79%, respectively.

Moving to MRI, in 2011, Zappa et al. [34] found a positive association between histopathological inflammation and MR-E signs, such as wall thickness, degree of wall enhancement on the delayed phase, pattern of enhancement, relative mural hyperintensity on T2-weighted sequences, loco-regional hypervascularity, presence of a fistula and abscesses. Moreover, the authors found the presence of fibrosis to correlate well with active inflammation, indicating that both processes (i.e., acute inflammatory infiltrate and apposition of fibrotic tissue) might be contemporarily present: based on these findings, they suggest that the dichotomous distinction between ‘inflammatory’ and ‘fibrotic’ patients might not be relevant in clinical practice.

In 2014, Tielbeek and al. [47] compared the histological scores of acute inflammation and fibrosis with the following MR-E findings: intestinal mural thickness measured on T2-weighted fat-saturated images, T1 and T2 ratio, maximum contrast enhancement and slope of increase after contrast injection. Mural thickness and T1 ratio correlated with both inflammation and fibrosis, likely owing to their simultaneous presence in the same bowel segment. A higher T2 ratio was significantly associated with more severe inflammation as well as with mild fibro-stenotic disease; conversely, a lower T2 ratio correlated with low inflammation scores and severe fibro-stenosis. Maximum enhancement and initial slope of increase showed a good correlation with histopathology, confirming the importance of intravenously administered gadolinium to assess disease activity. The same authors also assessed the role of diffusion-weighted imaging (DWI), a new sequence performed with multiple *b* values (usually 0–800 s/mm^2^ or 0–600 s/mm^2^) and a measurement of the signal in a manually drawn region of interest (ROI) placed on the bowel wall on the apparent diffusion coefficient (ADC) map. Notably, they observed a significant correlation between ADC decrease and fibrosis.

In 2015, Rimola et al. [48] compared three-grade inflammatory and fibrosis histopathological scores with the following MR-E findings: wall thickening, oedema, ulcers, signal intensity of the submucosa after injection gadolinium contrast medium at 70 s and 7 min, stenosis and contrast enhancement pattern. The authors found a correlation between inflammation and the presence of T2 hyperintensity, enhancement of the mucosa, presence of ulcers and blurred margin; on the other hand, the percentage of enhancement gain, pattern of enhancement at 7 min and presence of stenosis correlated with fibrosis. Finally, the percentage of enhancement gain could differentiate between mild-to-moderate and severe fibrosis (sensitivity 94%, specificity 89%).

More recently, Wilkens et al. [49,50] evaluated if there was a correlation between small bowel wall perfusion measurements and histopathological scores for inflammation or fibrosis in CD, using both ultrasound (US) and MR-E. It emerged that intestinal wall thickness, assessed with either US or MR-E, was a valid marker of inflammation, but not of fibrosis. Moreover, a relative contrast enhancement in both techniques could not differentiate between acute inflammatory and fibrosis.

Finally, Cicero et al. [51] evaluated 59 patients, further divided into non-surgical (never undergone surgery) and surgical (at least one surgical operation for CD). Signal intensity in DWI images was measured at the highest b-value within pathologic intestinal walls and at lymph nodes, spleen and psoas muscle to calculate relative ratios (bowel/spleen, bowel/psoas and bowel/lymph node). In the non-surgical group, a positive correlation was found between endoscopic activity (assessed by the Simple Endoscopic Score for Crohn’s Disease [50]) and all ratios; in the surgical group, endoscopic activity positively correlated only with the bowel/lymph node ratio and bowel/psoas ratio.

## 7. How Does Imaging Help Defining Disease Extension and Localization?

As previously reported, the length of the small bowel varies in relation to the age, sex, weight and height of the patient. Patients with CD seem to have a shorter small bowel than the general population; one of the explanations could be the presence of increased contractile activity of fibroblasts in the extracellular matrix in patients affected by stricturing CD [52,53].

Currently, the length of the small intestine is measured with MR-E or CT-E [54,55,56,57]. In 2014, Sinha et al. assessed the length of the small intestine with MR images using vascular imaging software, finding a good correlation between the MR-E and surgical measures [55].

Similar findings emerged in another study, in which the measures of 54 consecutive patients undergoing ileo-colic resection, calculated with CT-E or MR-E through 2D multiplanar (MPR) reconstructions, were compared with surgical results. The best correlation between the two measures emerged when the length of the pathological segments was less than or equal to 20 cm. For more extensive diseases, the imaging tends to overestimate the length of the pathological intestine [57].

In our department, we use postprocessing technologies, such as MPR, and specific post-processing 2D and 3D software (Vue PACS Carestream) to identify the small bowel and manually measure the pathological loops and residual normal bowel [54].

Measuring intestinal length is also useful in the presence of fistulas or stenosis: they can be precisely localized based on their distance from the ileocecal valve or from the Treitz ligament, thereby providing useful information to plan surgery or endoscopic dilation.

**Figure 19:**
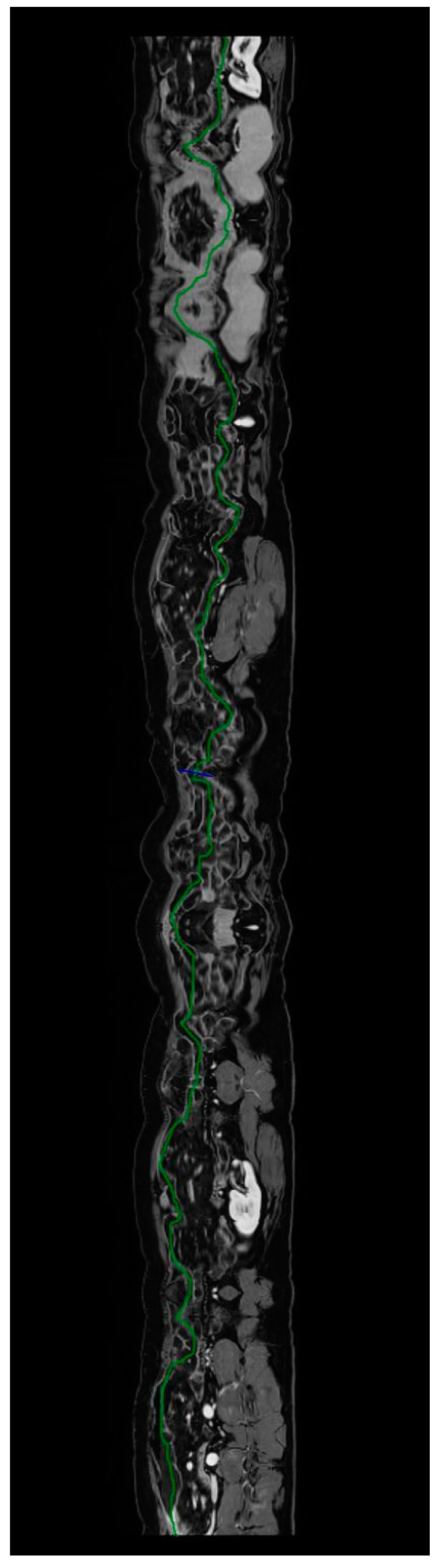
Calculation of the length of unaffected intestine using postprocessing software; a tubular view of the small bowel loops from MR-E is shown. The green line indicates the center of the bowel lumen. Original figures by LMM and LL.

**Figure 20:**
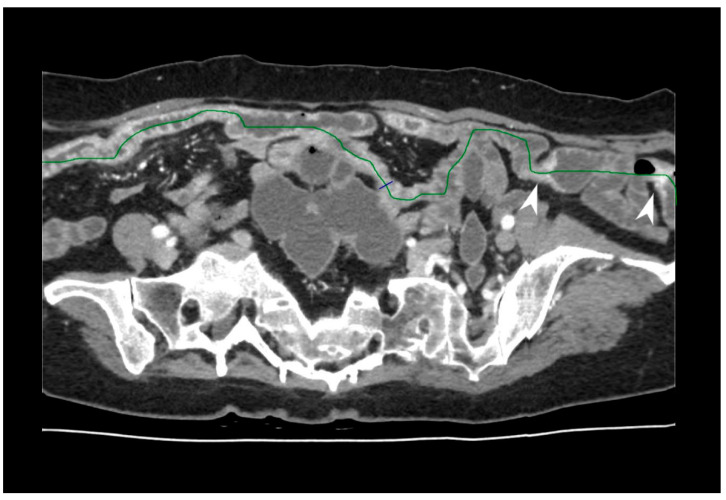
Calculation of the length of affected intestine using postprocessing software; a tubular view of the small bowel loops from CT-E is shown. There is evidence of two areas of pathological wall thickening on the right of the picture (arrowheads). The green line indicates the center of the bowel lumen. Original figures by LMM and LL.

Examples of measurement are shown in Figure 19 and Figure 20.

## 8. Can Radiological Imaging Assess Disease Activity?

Clinical manifestations of CD are quite variable, and they do not always correlate with the severity of endoscopic lesions or radiologic involvement. The Crohn’s Disease Activity Index (CDAI) is the most used clinical score in randomized controlled trials, but the complexity of its calculation, requiring a recall of 7 days, and the poor correlation with endoscopy limit its applicability in routine clinical practice [50]. The Harvey–Bradshaw Index represents an easier way to calculate clinical disease activity and has therefore been adopted in several real-world studies, but it has never been validated [50]. More recently, the Patient Reported Outcome (PRO)-2 score, including the two items of abdominal pain and stool frequency, is increasingly used in both clinical practice and clinical trials [58]. Biochemical parameters, mainly serum C-reactive protein and faecal calprotectin, are useful inflammatory markers for patient monitoring, and their normalization is currently considered an optimal target to pursue in the medium-term [59]. With regard to imaging, MR-E or CT-E are used to assess disease activity and also to evaluate how patients respond to medical therapy. Several scores have been developed so far to quantify the radiological activity of CD, showing a good correlation (Table 2) with clinical, laboratory and/or endoscopic parameters [31].

In a recent study comparing the MaRIA, Clermont and London indexes, the MaRIA index was shown to be the most accurate for the evaluation of disease activity and to grade its severity. The cut-offs established for the identification of active disease were 7 for the MaRIA, 8.4 for the Clermont index and 4.1 for the London index, while the cut-offs for severe inflammation were 11 for the MaRIA and 12.5 for the Clermont index [63].

Recently, Rimola et al. [64] evaluated the role of ADC values for the identification of bowel inflammation and therapeutic response in patients with CD treated with biologic therapy. The assessment of MaRIA score and the presence of endoscopic ulcers were determined at baseline and 46 weeks after starting therapy. Their findings did not support use of ADC rather than MaRIA scores for detecting the response to biologic therapy.

## 9. Is It Possible to Assess Post-Operative Recurrence?

About 20% of patients affected by CD undergo surgery in the first five years following the diagnosis and most of them experience post-operative recurrence (POR) [65]. Smoking habits, fistulising disease at index surgery, history of previous intestinal surgery and perianal disease are considered the main risk factors of POR. In the case of POR, endoscopic lesions usually precede the onset of symptoms [66]. Accordingly, in clinical practice, tight control with an endoscopy at 6–9 months after surgery and a stepwise treatment in the case of POR is routinely performed [67]. Currently, the endoscopic Rutgeerts score [50], albeit not validated, represents the reference standard scoring system in post-operative settings.

Several radiological studies have explored the role of MR-E or CT-E (± water enema) in comparison to endoscopy for detecting POR [68,69,70,71,72,73,74].

In particular, MR-E performed similarly to ileocolonoscopy for predicting clinical recurrence and to faecal calprotectin for predicting endoscopic recurrence [72]. Djelouah et al. [71] compared the diagnostic capabilities of MR-E using contrast-enhanced sequences to DWI, with endoscopy as the reference standard, and found that DWI-MRE has diagnostic capabilities similar to those of CE-MRE for the diagnosis of anastomotic POR. Schaefer et al. [68] developed and validated a MR imaging-based index (MONITOR index) to predict clinical POR and found that it was an efficient and reliable tool that can be used in clinical practice.

Choi et al. [70] evaluated the diagnostic yield and accuracy of CT-E and found that it can represent a viable option for early (<12 months) surveillance of anastomotic recurrence. Furthermore, the addition of a water enema provided a good distension of both sides of ileocolic anastomoses, allowing for the detection of recurrence [69].

## 10. Conclusions

CD is characterized by transmural inflammation potentially involving any tract of the digestive tube; the progression of disease can induce the development of complications and irreparable bowel damage. Imaging has a key role in clinical practice for assessing disease activity and extension and characterizing the disease pattern (i.e., inflammatory, fibrotic or fistulizing/penetrating). All these aspects have a meaningful impact on patient management, especially in those situations requiring a precise risk–benefit assessment between medical and surgical approaches. Moreover, imaging can be used for detecting POR, even though endoscopy still represents the reference standard. CT and MRI can both answer the abovementioned questions, but MRI should be preferred, owing to its greater sensitivity and specificity for assessing disease activity and to the lack of ionizing radiation. Regardless of the technique employed, adequate distension of the small bowel is essential to maximize its diagnostic performance, while the distention of the colon, via rectal enemas, should be adopted only in special situations.

In conclusion, radiologists are essential healthcare professionals to be involved in multidisciplinary teams for the management of CD patients, and strict collaboration is required among specialists to obtain the necessary answers for clinically relevant questions.

Our review aimed to be as pragmatic as possible by answering clinical questions that are posed daily in the management of CD, and it has provided a synthesis of the available data on these topics; however, a systematic review was not performed, and this review might suffer from subjectivity in the determination of which studies to include, the way the studies were analysed and the conclusions drawn.

Despite providing some useful indications regarding the role of radiology in the management of patients with CD, further research and systematic reviews are undoubtedly needed.

## Figures and Tables

**Table 1:** Types of oral contrast agents and their advantages and disadvantages.

Oral Contrast Agents	Advantages	Disadvantage
Water	Well tolerated, biphasic properties in MRI	Rapid absorption
PEG	Better distension than water and MC	The least tolerated of all agents due to diarrhoea
MC	Better tolerated than PEG	Lower distension than PEG and dilute barium sulphate
dilute barium sulphate	Good distension	Mild side effects (gas and diarrhoea)
Oil emulsions	Grater viscosity than water	Little tolerated

PEG, polyethylene glycol solution; MC, methylcellulose.

**Table 2:** (**a**) MEGS and CDMI. (**b**) MARIA Score and Clermont index.

(**a**)		
	**MEGS Score (Score from 0 to 3)**	**CDMI Score (Score from 0 to 3)**
Mural thickness of small bowel	0: <3 mm 1: >3–5 mm 2: >5–7 mm 3: >7 mm	0: <3 mm 1: >3–5 mm 2: >5–7 mm 3: >7 mm
Mural T2 signal compared to normal bowel wall	0: Equivalent to normal bowel wall 1: Minor increase in signal on fat-saturated images 2: Moderate increase in signal. 3: Marked increase in signal	0: Equivalent to normal bowel wall 1: Minor increase in signal on fat-saturated images 2: Moderate increase in signal. 3: Marked increase in signal
Perimural T2 signal	0: Equivalent to normal mesentery 1: Increase in mesenteric signal but no fluid 2: Small fluid rim (≤2 mm) 3: Larger fluid rim (>2 mm)	0: Equivalent to normal mesentery 1: Increase in mesenteric signal but no fluid 2: Small fluid rim (≤2 mm) 3: Larger fluid rim (>2 mm
T1 enhancement compared with nearest vessels	0: Equivalent to normal bowel wall 1: Minor enhancement of the bowel wall 2: Moderate enhancement but somewhat less than the nearby vascular structures 3: Moderate enhancement similar to the nearby vascular structures	0: Equivalent to normal bowel wall 1: Minor enhancement of the bowel wall 2: Moderate enhancement but somewhat less than the nearby vascular structures 3: Moderate enhancement similar to the nearby vascular structures
Mural enhancement pattern	0: Not available or homogeneous 1: Mucosal 2: Layered	0: Not available 1: Homogeneous 2: Mucosal 3: Layered
Haustral loss	0: None 1: <1/3 segment 2: 1/3 to 2/3 segment 3: >2/3 segment	
Multiplication factor per segment Length of disease segment	0–5 cm × 1 5–15 cm × 1.5 >15 cm × 2	
Additional score for extramural features	0–5	3 nodes greater than 1 cm
Lymph nodes (short diameter 1 cm or more)	Absent, present	0: absent 1: Cluster less than 1 cm 2: 1 node greater than 1 cm 3: 3 nodes greater than 1 cm
Engorged vasa recta	Absent, present	Absent, present
Abscess	Absent, present	
Fistulae	Absent, present	
Other		Lymph node enhancement compared to nearest vessel; Less than nearby vascular structure; Equivalent or greater to nearby vascular structure
(**b**)		
	**MARIA Score**	**Clermont Index**
Bowel mural thickness	>3 mm	>3 mm
Presence of mucosal ulcers (deep grooves in the mucosa)	No, yes	No, yes
Presence of wall oedema (hyperintensity on T2-weighted images of the bowel mural layers relative to the signal of the psoas muscle)	No, yes	No, yes
Wall signal intensity (WSI)	WSI was calculated in the areas with the predominant thickening and corresponded to the average of three WSI measurements	Not evaluated
Relative contrast enhancement (RCE)	RCE = ((WSI post-gadolinium − WSI pre-gadolinium)/(WSI pre-gadolinium)) × 100 × (standard deviation-SD noise pre-gadolinium/SD noise post-gadolinium).	Not evaluated
Fistulas, abscesses, enlarged (>8 mm) regional mesenteric lymph nodes, and fibrofatty proliferation.	Not evaluated	No, yes
DWI hyperintensity	Not evaluated	No, yes

MEGS Score = (Jejunal Score × Factor for Jejunum Involved Length) + (Proximal Ileum Score × Factor for Proximal Ileum Length) + (Terminal Ileum Score × Factor for Terminal Ileum Length) + (Caecum Score × Factor For Caecum Length) + (Ascending Score × Factor for Ascending Length) + (Transverse Score × Factor for Transverse Length) + (Descending Score × Factor for Descending Length) + (Sigmoid Score × Factor for Sigmoid Length) + (Rectum Score × Factor for Rectum Length) + Score for Abscess + Score for Fistula + Score for Adenopathy + Score for engorged vasa recta. No cut-off [60]. MRI index: 1.79 + 1.34 mural thickness (mm) + 0.94 mural T2 score; cut off = 4.1 [61]. MaRIA = 1.5 × mural thickness (mm) + 0.02 × relative contrast enhancement + 5 × oedema + 10 × ulcers. The total MaRIA score was calculated as the sum of the MaRIA in each intestinal tract. The cut-off points established for differentiating active from inactive disease is 7. A maria score > 11 is highly predictive of severe ileal CD [31]. Clermont score: –1.321 × ADC (mm^2^/s) + 1.646 × mural thickening (mm) + 8.306 × ulcerations + 5.613 × oedema + 5.039. A Clermont score > 8.4 is highly predictive of ileal CD activity. A value of Clermont score ≥ 12.5 is highly predictive of severe ileal CD [62].

## Data Availability

No new data were created.
